# Beyond acronyms: a domain-based structural analysis of abdominal wall hernia repair techniques in the contemporary literature

**DOI:** 10.1007/s00464-026-13254-x

**Published:** 2026-08-04

**Authors:** Francesco Brucchi, Rita Stanco, Giampaolo Formisano, Paolo Pietro Bianchi, Maaike Vierstraete, Gianlorenzo Dionigi, Filip Muysoms

**Affiliations:** 1 Department of Pathophysiology and Transplantation, University of Milan, 20122 Milan, Italy; 2 Division of General Surgery, Istituto Auxologico Italiano, IRCCS (Istituto di Ricovero e Cura a Carattere Scientifco), Via Mercalli 30, Milan, Italy; 3 University of Pavia, Pavia, Italy; 4 Dipartimento Scienze Della Salute, Univesity of Milano, Milan, Italy; 5 Department of Plastic, Reconstructive and Aesthetic Surgery, ZAS Antwerp, Antwerp, Belgium; 6 Department of General Surgery, AZ Maria Middelares, Ghent, Belgium

**Keywords:** Abdominal wall hernia, Surgical technique, Nomenclature, Domain-based classification, Label redundancy, Operative domains, Mesh repair, Shannon entropy, Muysoms nomenclature

## Abstract

**Background:**

Surgical techniques in abdominal wall surgery are commonly described using labels, including acronyms, eponyms, and narrative descriptors, that only partially capture their technical characteristics. Whether these labels correspond to genuinely distinct procedures or instead represent variations within a smaller number of underlying technical configurations remains empirically untested.

**Methods:**

Building on our methodological study of reporting completeness, we performed a structured domain-based analysis of abdominal wall hernia repair techniques. Each surgical arm was decomposed into five predefined operative domains: surgical approach (A), hernia type (H), mesh position (M), mesh type (T), and fixation method (F). Unique technical configurations were defined as distinct domain combinations. Terminological redundancy was evaluated by mapping labels to their corresponding configurations. Technical diversity was quantified using Shannon entropy.

**Results:**

Two hundred studies met inclusion criteria, yielding 290 surgical arms. Domain decomposition identified 184 unique technical configurations. Among 170 unique labels, 21 (12.4%) mapped to more than one five-domain configuration. Conversely, among 93 fully specified configurations linked to at least one label, 21 (22.6%) were described by more than one label. The most polysemous label (TEP) corresponded to 24 distinct configurations. Groin hernia repairs accounted for the largest number of arms (*n* = 130) but showed moderate structural diversity relative to sample size, whereas ventral and incisional repairs demonstrated higher variability per arm. In the sensitivity analysis restricted to the 149 fully specified arms, 94 unique configurations were identified; polysemy and redundancy remained substantial (TEP mapped to 11 configurations).

**Conclusion:**

Abdominal wall hernia repair techniques are more completely represented when the labels in common use—acronyms, eponyms, and narrative descriptors—are supplemented by structured operative domains rather than relied upon in isolation. Domain-based descriptors provide a reproducible, machine-readable complement to existing nomenclature that may improve reporting clarity, support comparative research, and facilitate structured classification of surgical techniques.

**Supplementary Information:**

The online version contains supplementary material available at https://doi.org/10.1007/s00464-026-13254-x.

Clear, reproducible reporting of surgical techniques is a prerequisite for meaningful comparative research, reliable systematic reviews, and registry-based analyses. In abdominal wall surgery, however, operative procedures are typically identified by labels—a term used here to encompass acronyms (e.g., TEP, TAPP, IPOM), eponyms (e.g., Lichtenstein, Rives), and narrative descriptors (e.g., “open retromuscular mesh repair”)—that capture only a fraction of the technical information required to define a procedure [1, 2]. The rapid proliferation of minimally invasive and robotic approaches has further expanded this terminological landscape, creating a growing gap between the labels used to name procedures and the operative details they actually convey [3].

Previous work has highlighted the uncontrolled growth of acronyms in hernia surgery and proposed rationalized nomenclature systems [1, 2, 4]. Despite these efforts, no empirical study has systematically evaluated whether commonly used labels correspond to technically distinct entities or instead represent variable combinations of a smaller number of underlying procedural components.

An alternative conceptual framework treats surgical procedures not as monolithic entities defined by their names, but as modular combinations of fundamental operative domains. In abdominal wall surgery, these domains include the surgical approach, the type of hernia repaired, the anatomical plane of mesh placement, the prosthetic material used, and the fixation strategy employed [2]. Under this model, each procedure becomes a unique coordinate in a multidimensional technical space, and the relationship between labels and procedures becomes an empirically testable question.

The present study aimed to characterize the contemporary technical landscape of abdominal wall hernia repair by decomposing procedures reported in the literature into standardized operative domains and quantifying both their structural diversity and the degree of terminological redundancy associated with label-based reporting.

## Materials and methods

### Study design and terminological conventions

This was a descriptive methodological study designed to characterize the technical landscape of contemporary abdominal wall surgery literature using structured operative domains. These five domains correspond exactly to the Muysoms A–H–M–T–F nomenclature (Approach–Hernia–Mesh position–Mesh type–Fixation) recently proposed for standardized reporting [2]. The study did not assess clinical outcomes, comparative effectiveness, or patient-level data.

Throughout this report, the term “label” is used as an umbrella term to denote any identifier applied to a surgical technique in the source literature, including formal acronyms (e.g., TEP, TAPP), eponyms (e.g., Lichtenstein), and narrative descriptors (e.g., “open retromuscular mesh repair”). The term “acronym” is reserved for the subset of labels that take an abbreviated form. This distinction is adopted because the dataset includes both types and the analysis addresses terminological redundancy at the label level rather than exclusively among acronyms.

The primary objective was to determine the number and distribution of unique technical configurations identifiable when procedures reported in the literature are decomposed into standardized domains. Secondary exploratory objectives included quantifying label–configuration discordance and assessing structural diversity across hernia types.

Although the study shares features with a scoping review in terms of breadth and descriptive intent, the unit of analysis was the individual surgical technique rather than the publication, and the research question concerned the internal structural composition of procedures rather than the mapping of a research field. Reporting was informed by the guideline for meta-epidemiological methodology research proposed by Murad and Wang, adapted to the descriptive nature of the present analysis [5]. The study design, technical domains, outcome definitions, and analytical framework were established a priori before data extraction. No formal protocol was prospectively registered, as protocol registration platforms are designed for systematic reviews of clinical questions and are not applicable to this study type.

### Literature search and study selection

A structured literature search was performed in PubMed/MEDLINE to identify original studies reporting surgical techniques for abdominal wall hernia repair. The search covered January 2000 to November 2025. The following search strategy was used, combining free-text terms and MeSH headings:

*(“hernia repair” OR “abdominal wall” OR inguinal OR ventral OR incisional OR parastomal) AND (laparoscopic OR robotic OR open OR endoscopic) AND (technique OR approach OR mesh OR repair)*

The dataset derives from the same literature corpus examined in our previous methodological study of reporting completeness; the same search strategy, screening logic, and concept of descriptive saturation were maintained to ensure methodological continuity [4].

Titles and abstracts were screened independently by two reviewers. (RS, FB) Full-text articles were assessed for eligibility. Disagreements were resolved by consensus. Screening continued until descriptive saturation was reached, operationally defined as the point at which inclusion of additional consecutive batches of 20 studies did not result in meaningful changes in the overall distribution of reporting completeness rates across the five predefined technical domains.

Studies were included if they involved adult patients; addressed abdominal wall hernia repair (inguinal, ventral, incisional, spigelian, umbilical, or parastomal); reported a defined surgical technique using an open, laparoscopic, robotic, or extraperitoneal endoscopic approach; and were published within the predefined time frame. Studies were excluded if they were narrative reviews, systematic reviews, or meta-analyses; case reports, technical notes without outcome data, or video-only publications; or lacked any meaningful technical description of the surgical procedure.

### Unit of analysis

The unit of analysis was the surgical technique rather than the individual publication. When a study reported more than one surgical approach or technique, each was considered a separate surgical arm and analyzed independently.

### Definition of technical domains

**Fig. 1:**
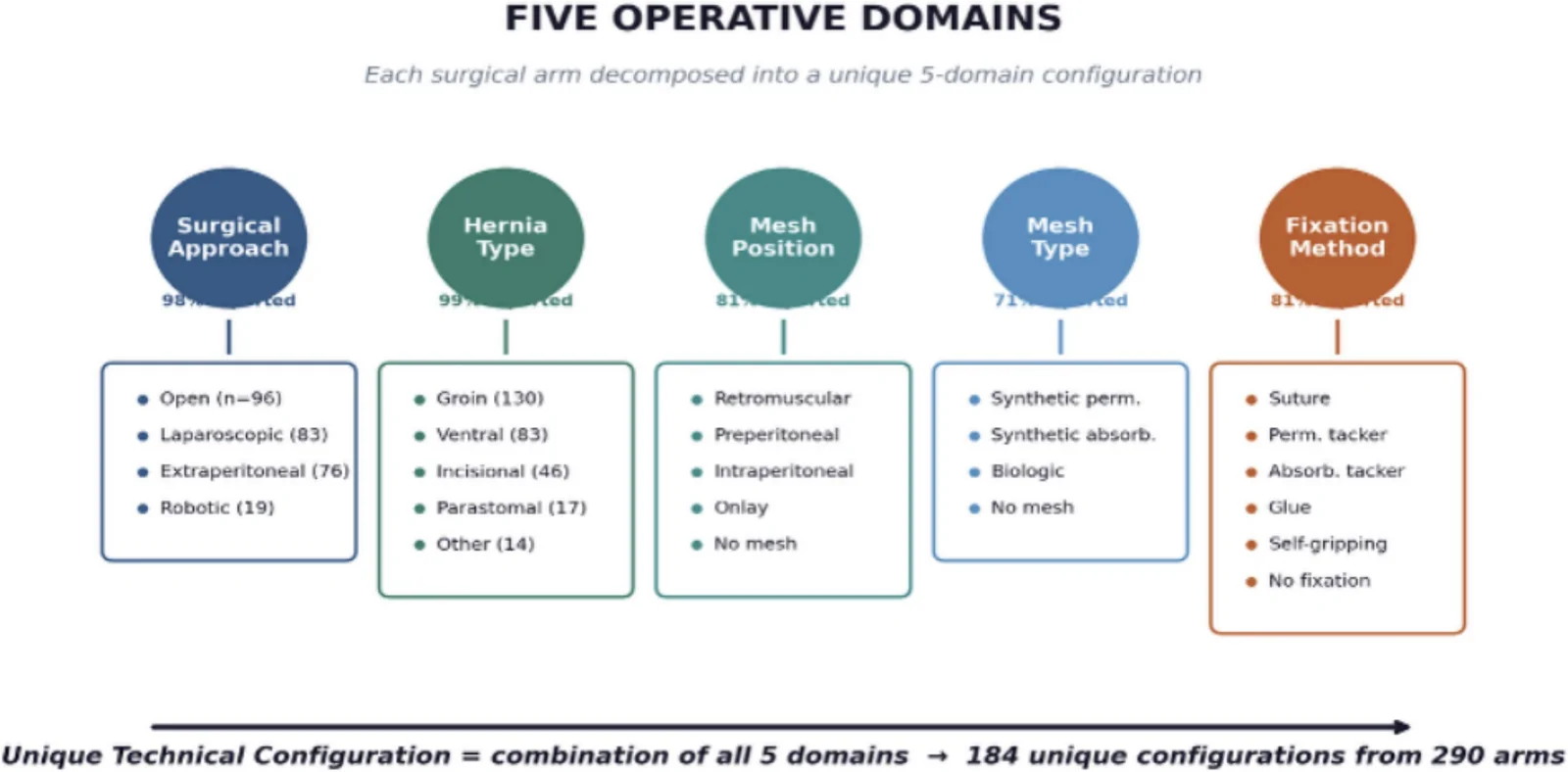
Conceptual framework: five operative domains used for structured decomposition of surgical techniques. Percentages indicate domain-level reporting completeness across the dataset

Each surgical arm was evaluated across five predefined technical domains, selected a priori as essential descriptors of procedural structure in abdominal wall surgery: surgical approach, hernia type, mesh position, mesh type, and fixation method (Fig. 1). Domains were considered reported only when explicitly stated in the manuscript. Information inferable by expert interpretation but not explicitly described was classified as not reported.

These five domains were selected as a pragmatic first-step set of core technical elements sufficient to capture the structural identity of a surgical procedure for large-scale literature analysis. Additional outcome-relevant factors—such as defect size, fascial closure technique, component separation details, and dissection extent—were deliberately excluded as secondary modifiers that would have substantially increased the complexity of the assessment framework without altering the primary structural analysis.

### Technical configurations and label mapping

Surgical techniques sharing identical five-domain combinations were grouped. For each unique configuration, the labels used in the literature were recorded. Label–configuration discordance was examined bidirectionally: first, by identifying fully specified configurations associated with more than one label (label redundancy); and second, by identifying labels that mapped to more than one configuration (label polysemy). Redundancy analyses were restricted to fully specified configurations (i.e., those with no missing domain values) for which at least one explicit label was available. This analysis was exploratory and was not intended as a systematic linguistic assessment.

### Sensitivity analysis

A pre-specified sensitivity analysis was restricted to the 149 fully specified surgical arms (all five domains explicitly reported; 51.4% of the original sample). In this subgroup, the number of unique technical configurations, label polysemy, label redundancy, and Shannon entropy were recalculated using the same methods.

### Data extraction and reviewer roles

Data extraction was performed using a predefined data dictionary. Initial extraction was conducted by a reviewer without formal training in abdominal wall surgery, focusing on identification of reported technical elements. This approach minimized expert-driven interpretation. A second reviewer with specific expertise and dedicated fellowship training independently reviewed all extracted data. Discrepancies were resolved through adjudication by a senior expert (FM).

### Outcomes

The primary outcome was the number and distribution of unique technical configurations identified after decomposition of each surgical arm into five operative domains. Secondary outcomes included the degree of label–configuration discordance; technical diversity across hernia types, quantified using Shannon entropy; the distribution of procedures across the five predefined domains; and domain-level reporting completeness rates.

### Statistical analysis

Analyses were descriptive. Results were summarized using counts and percentages. Technical diversity was quantified using Shannon entropy, calculated as *H* = − Σ *p*i log₂ (*p*i), where pi represents the proportion of surgical arms assigned to each unique configuration within a hernia type [6]. Higher entropy values indicate greater dispersion across configurations; lower values indicate concentration around fewer dominant techniques. No formal hypothesis testing was planned.

### Ethics

This study was based exclusively on published literature and did not involve human participants or individual patient data. Formal ethical approval was not required.

## Results

### Study sample

**Fig. 2:**
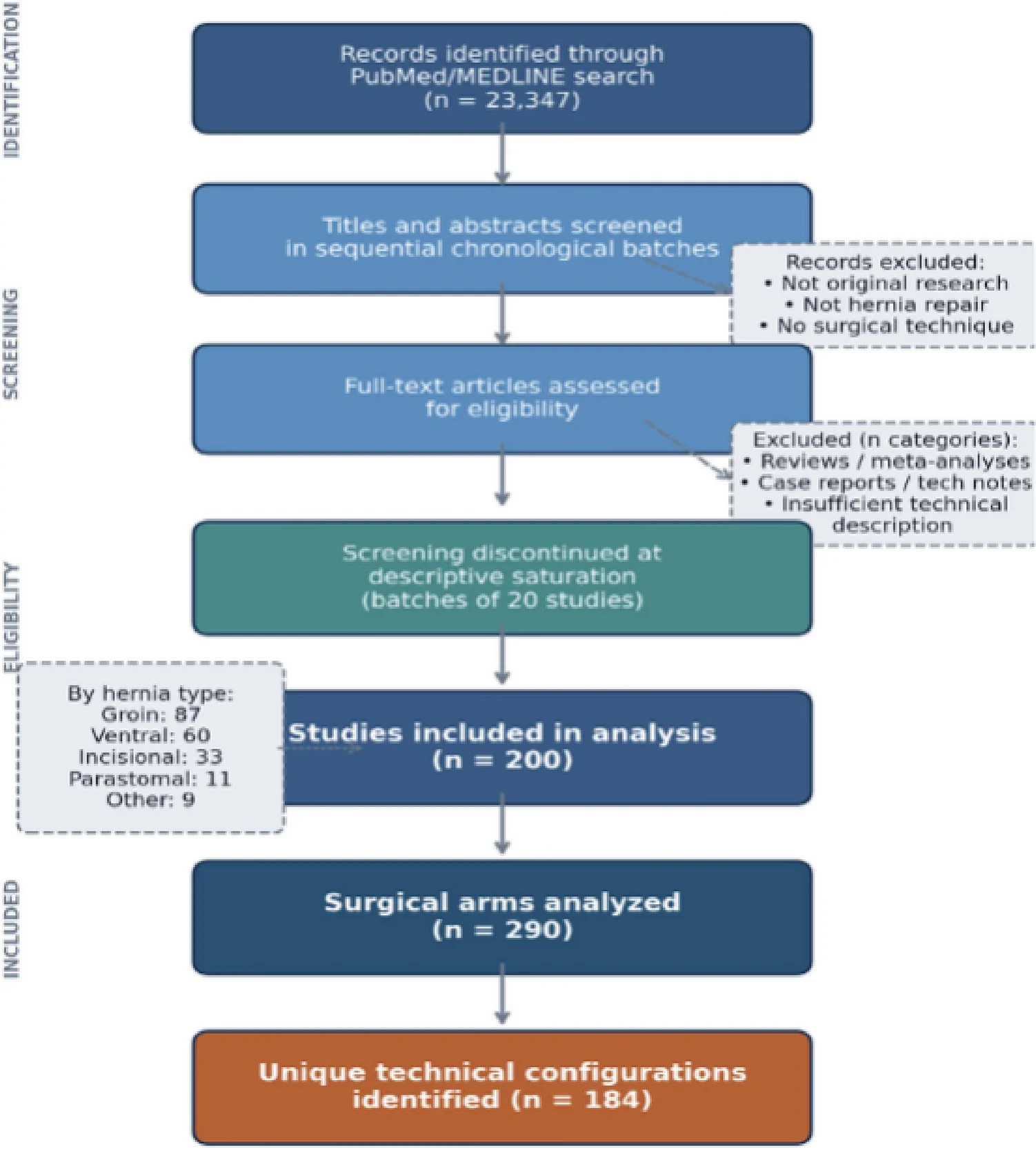
Study selection flowchart

The PubMed search yielded 23,347 records. Titles and abstracts were screened sequentially in chronological batches until descriptive saturation was reached (Fig. 2). After full-text assessment and application of inclusion and exclusion criteria, 200 studies were retained [1, 7–204]. The main reasons for exclusion were: narrative or systematic reviews and meta-analyses; case reports or technical notes without outcome data; and insufficient technical description of the operative procedure. Because several included studies reported more than one surgical technique, the final dataset comprised 290 distinct surgical arms.

At the study level, the included articles addressed groin hernias (87 studies), ventral hernias (60 studies), incisional hernias (33 studies), parastomal hernias (11 studies), and other types (9 studies). At the arm level, groin hernia repairs constituted the largest group (130 arms), followed by ventral (83 arms), incisional (46 arms), parastomal (17 arms), umbilical (7 arms), spigelian (2 arms), and other types (5 arms). The surgical approach was open in 96 arms, laparoscopic in 83, extraperitoneal endoscopic in 76, and robotic in 19.

### Domain reporting completeness

**Fig. 3:**
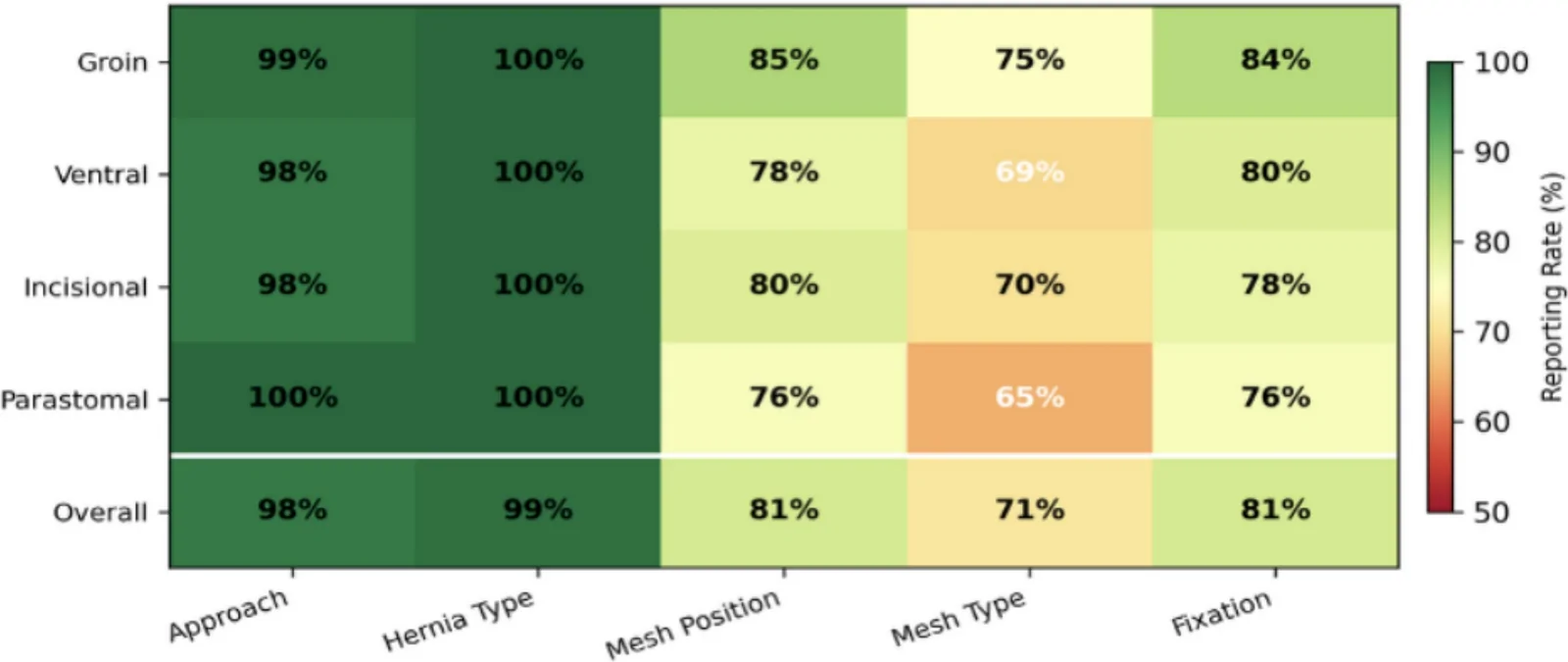
Domain reporting completeness across hernia types. Values represent the percentage of arms in which each domain was explicitly reported

Reporting completeness varied substantially across domains (Fig. 3). Surgical approach and hernia type were reported in nearly all arms (98.3% and 99.3%, respectively). In contrast, mesh position was explicitly reported in 80.7% of arms, fixation method in 81.4%, and mesh type in only 71.4%. This pattern of incomplete specification was consistent across hernia types, although parastomal hernia repairs showed the lowest completeness for mesh type (65%).

### Structural diversity of technical configurations

Decomposition of the 290 surgical arms into five operative domains identified 184 unique technical configurations, corresponding to an average of 1.6 arms per configuration (Fig. 1). This finding indicates substantial structural heterogeneity in the way abdominal wall hernia repair techniques are represented in the literature.

### Variation across hernia types

**Fig. 4:**
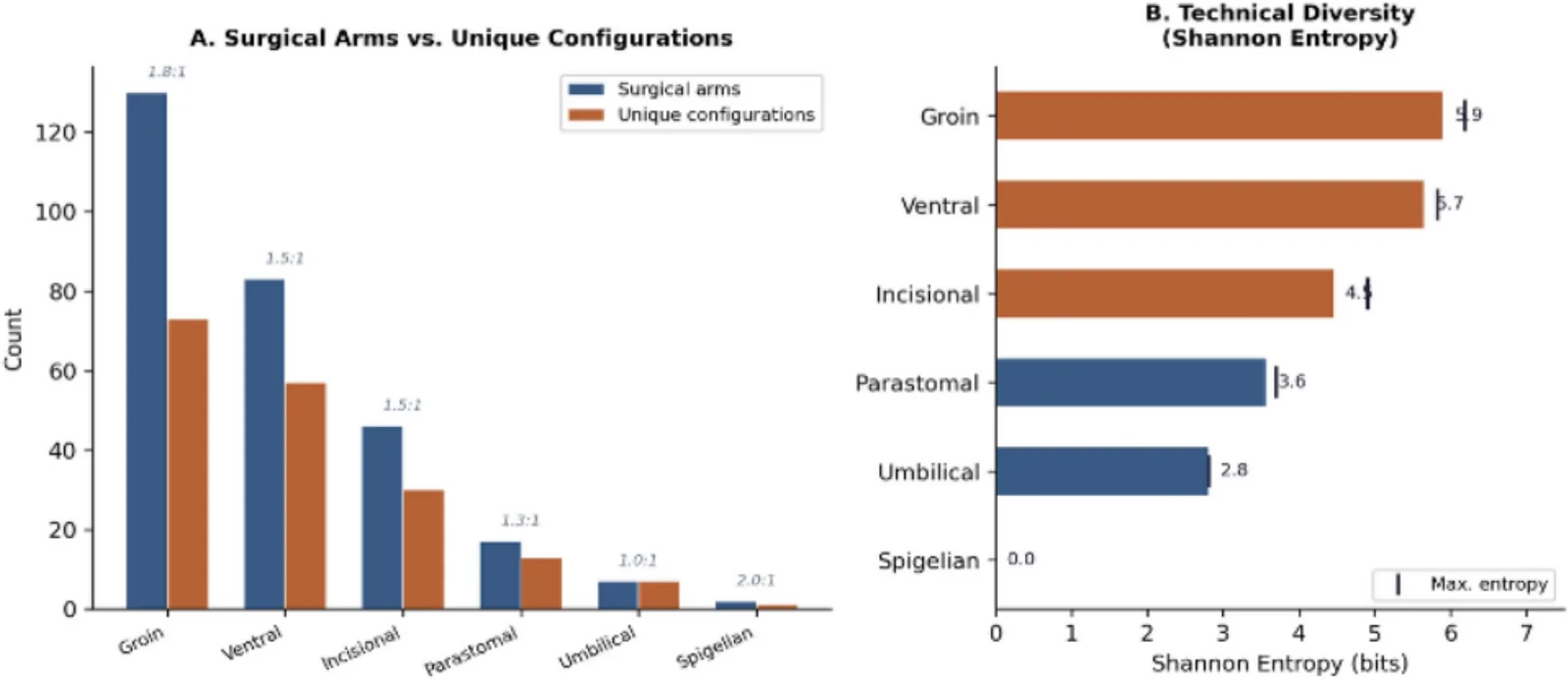
Structural diversity of technical configurations across hernia types. **A** surgical arms versus unique configurations. **B** Shannon entropy (observed vs. theoretical maximum)

Structural diversity varied according to hernia type (Fig. 4). Groin hernia repairs, despite accounting for the largest number of arms (*n* = 130), yielded 73 unique configurations (ratio 1.8:1), suggesting moderate procedural convergence. Ventral and incisional hernia repairs demonstrated higher variability per arm (57 configurations from 83 arms [ratio 1.5:1] and 30 configurations from 46 arms [ratio 1.5:1], respectively), reflecting differences in defect characteristics, anatomical complexity, and patient-specific factors. Shannon entropy confirmed this gradient: groin repairs showed the highest absolute entropy (*H* = 5.90 bits) due to the large number of configurations, while ventral (*H* = 5.65) and incisional repairs (*H* = 4.46) approached their theoretical maxima more closely, indicating a more even distribution of arms across configurations.

### Label–configuration discordance

**Fig. 5:**
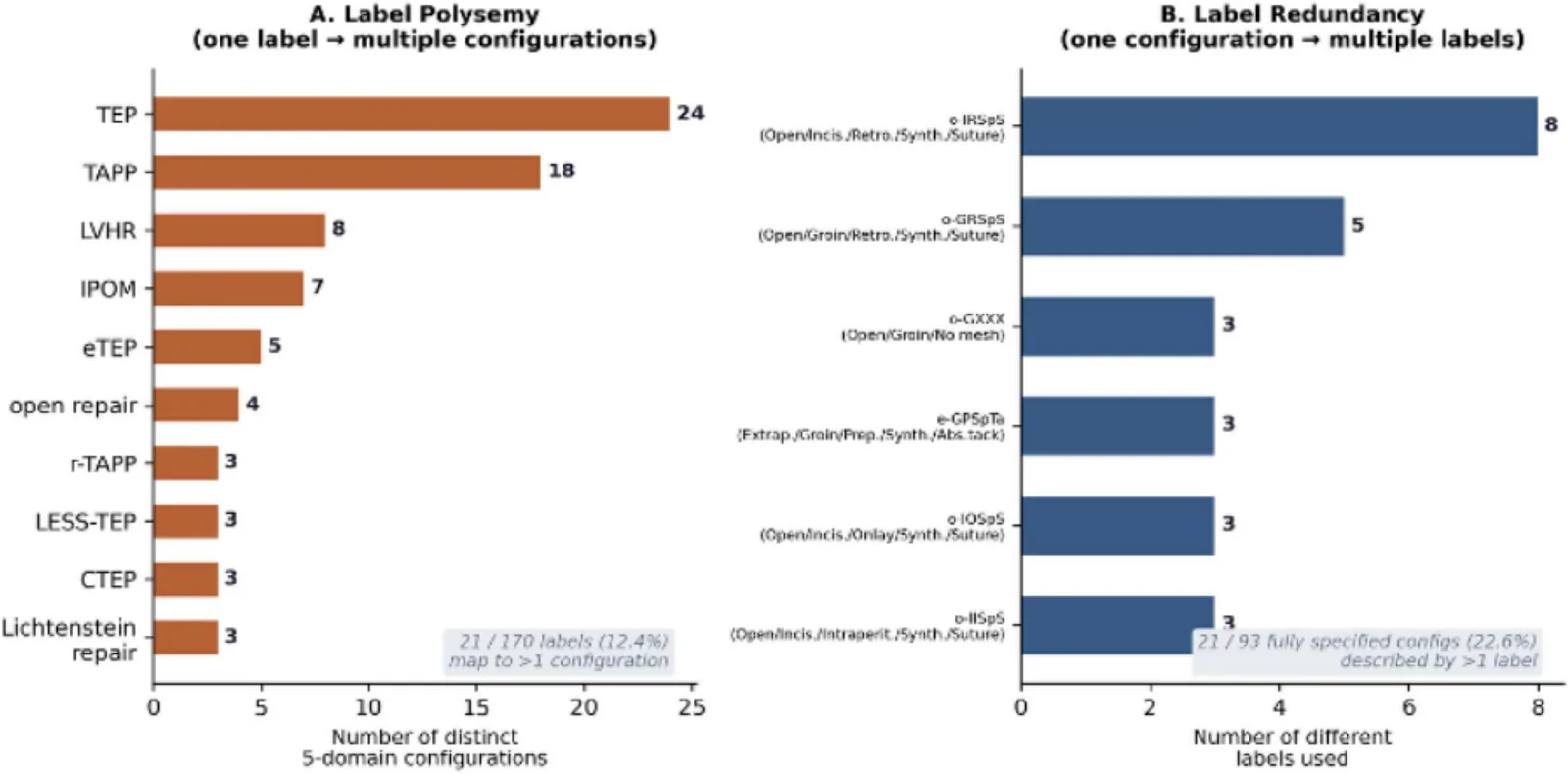
Label–configuration discordance. **A** labels mapping to multiple five-domain configurations (polysemy). **B** fully specified configurations described by multiple labels (redundancy)

Mapping labels to domain-defined configurations revealed substantial terminological redundancy in both directions (Fig. 5).

#### Label polysemy

Among 170 unique labels identified in the dataset, 21 (12.4%) mapped to more than one five-domain configuration. The most polysemous label was TEP, which corresponded to 24 distinct configurations—largely because source reports variably specified or omitted the mesh type and fixation domains. TAPP mapped to 18 configurations, LVHR to 8, and IPOM to 7. Complete configuration lists for polysemous labels are provided in Supplementary Table S1.

#### Label redundancy

Among the 184 unique configurations, 93 were both fully specified (no missing domain values) and linked to at least one explicit label. Within this subset, 21 (22.6%) were described by more than one label. The highest degree of redundancy was observed for the configuration o-IRSpS (open, incisional, retromuscular, synthetic permanent mesh, suture fixation), which was described using 8 different labels in the literature, including “sublay technique,” “open Rives retromuscular repair,” and “open incisional hernia repair.” Similarly, the Lichtenstein configuration (o-GRSpS) appeared under 5 distinct labels. Representative examples are shown in Table 1.

**Table 1:** Representative examples of label–configuration discordance

Panel A. Multiple labels for one fully specified 5-domain configuration		
Domain code	5-domain configuration	Labels used in literature
o-IRSpS	Open; Incisional; Retromuscular; Synthetic perm.; Suture	LIH repair; SIH hernia open surgical repair; open Rives retromuscular repair; open incisional hernia repair; open repair; open retromuscular LIH repair; open retromuscular mesh repair; sublay technique
o-GRSpS	Open; Groin; Retromuscular; Synthetic perm.; Suture	Lichtenstein procedure; Lichtenstein repair; open tension-free hernia repair; OLR
o-GXXX	Open; Groin; No mesh; No mesh; No fixation	Moloney darn; herniotomy; Desarda technique
o-IOSpS	Open; Incisional; Onlay; Synthetic perm.; Suture	Open repair; onlay mesh repair; onlay technique
o-IISpS	Open; Incisional; Intraperitoneal; Synthetic perm.; Suture	Open incisional hernia repair; open IPOM; large incisional hernia open repair

Panel B. One label for multiple 5-domain configurations (summary; see Supplementary Table S1 for complete configurations)		
Label	Nature of configuration variation	N Configs
TEP	All extraperitoneal groin repairs; variation primarily in mesh type and fixation domain specification	24
TAPP	All laparoscopic groin preperitoneal repairs; variation in mesh type (synthetic, biologic, absorbable) and fixation (glue, tacker, self-gripping, suture, none)	18
LVHR	All laparoscopic ventral repairs; variation in mesh position (intraperitoneal vs. unspecified) and fixation method	8
IPOM	Mixed approaches (laparoscopic, open) and hernia types (ventral, spigelian); variation across all downstream domains	7
eTEP	Extraperitoneal approach for both groin and ventral hernias; retromuscular mesh position; variation in mesh type and fixation	5

*LIH* large incisional hernia, *SIH* suprapubic incisional hernia, *OLR* open Lichtenstein repair

In Panel B, some apparent label–configuration multiplicity reflects incomplete domain specification in source reports rather than true semantic polysemy. Supplementary Table S1 provides the complete list of configurations for each polysemous label

Overall, these findings demonstrate that label-based nomenclature frequently fails to provide a unique or reproducible identifier of surgical technique when procedures are evaluated through structured operative domains.

### Sensitivity analysis restricted to fully specified arms

When the analysis was limited to the 149 arms with complete five-domain specification, 94 unique technical configurations were identified (versus 184 in the full dataset). Label polysemy remained substantial: 15 of the 142 unique labels (10.6%) mapped to more than one configuration, with TEP corresponding to 11 distinct configurations and TAPP to 12. Among the fully specified configurations linked to at least one label, 17 (22.6%) were described by more than one label. Shannon entropy values were moderately lower but preserved the same gradient across hernia types (groin: 5.04 bits; ventral: 4.49 bits; incisional: 3.17 bits). These findings confirm that the observed structural diversity and terminological redundancy are not merely artifacts of incomplete reporting.

## Discussion

This study provides a structural analysis of how surgical techniques are reported in the abdominal wall hernia repair literature. By decomposing procedures into fundamental operative domains, the analysis demonstrates that the apparent proliferation of named techniques largely reflects combinations of a limited number of recurring technical elements rather than a genuine expansion of procedurally distinct operations.

The findings reveal considerable heterogeneity: from 290 surgical arms, domain decomposition identified 184 unique configurations. This observation underscores both the complexity of abdominal wall surgery and the wide spectrum of technical variations currently described in the literature.

A clear contrast emerged between hernia types. Groin hernia techniques yielded a ratio of 1.8 arms per configuration, suggesting moderate convergence toward shared procedural patterns—consistent with progressive standardization through decades of randomized trials and guideline development. Ventral and incisional hernia repairs, by contrast, demonstrated ratios closer to 1.5:1, reflecting the inherent heterogeneity of these conditions in terms of defect size, location, contamination status, and patient comorbidity. Ventral and incisional repairs were also spread more evenly across their configurations than groin repairs, indicating that these fields still lack the dominant procedural standards that decades of randomized trials have produced for groin hernia repair.

Perhaps the most consequential finding is the degree of label–configuration discordance. TEP mapped to 24 distinct configurations and TAPP to 18—observations that raise fundamental questions about what is actually being compared when these labels are listed as study interventions or included in meta-analyses. The implications extend beyond semantic imprecision. At each of these levels the same difficulty recurs: when one label denotes many distinct procedures, a reader cannot reliably interpret what was actually done from the name alone; two studies cannot be assumed to have evaluated the same intervention merely because they share a label; and the reported technique fails to convey the detail a later analyst would need in order to reconstruct or pool it. When two studies both report “TEP” but one involves a self-gripping polypropylene mesh with no additional fixation while the other uses a permanent mesh with tacker fixation, the apparent homogeneity of the label masks substantive technical differences that may influence outcomes. Label-based reporting does not merely create linguistic inconsistency; it introduces a risk of procedure-level misclassification with direct consequences for study comparability, data pooling, and the validity of secondary analyses [1, 2].

Importantly, not all label–configuration multiplicity should be interpreted as true semantic polysemy. In a substantial proportion of cases, discordance reflects incomplete specification of one or more operative domains in the source reports, so that the same label appears linked to partially specified rather than genuinely distinct configurations. The reporting completeness analysis (Fig. 3) supports this interpretation: while surgical approach and hernia type were reported in nearly all arms (> 98%), mesh type was explicitly reported in only 71.4% and fixation method in 81.4%. This observation does not diminish the methodological concern—whether arising from true technical variation or incomplete domain specification, label-based reporting still introduces classification uncertainty at the level of procedure definition.

The sensitivity analysis restricted to the 149 fully specified arms confirmed that the high degree of structural heterogeneity and terminological redundancy persists even when incomplete domain specification is eliminated. This strengthens the conclusion that label-based nomenclature is inherently imprecise, independent of reporting quality.

Domain-based procedural representation may offer a pragmatic alternative. The five-domain model proposed here is deliberately parsimonious and can be expressed as a compact alphanumeric string (e.g., o-IRSpS for an open incisional retromuscular repair with synthetic permanent mesh and suture fixation). Such notation is simultaneously human-readable and machine-parseable, making it suitable for structured reporting in clinical trials, integration into surgical registries, and automated classification in large datasets. To make this concrete, the twenty-four configurations currently recorded under the single acronym “TEP” could each be stored in a hernia registry (for example, EuraHS) as a distinct A–H–M–T–F string, so that a query for a particular mesh type and fixation combination returns only technically comparable procedures rather than every record sharing the label. In a clinical-trial report, the same string (e.g., o-IRSpS) could populate a structured “intervention” field alongside the conventional name, allowing the exact technique to be extracted electronically without manual re-reading of the operative methods. In a systematic review, eligible arms could then be filtered and pooled by exact domain configuration rather than by shared label, ensuring that only genuinely equivalent techniques are combined within a single meta-analytic comparison. Future iterations may extend the model to include additional modifiers such as fascial closure technique, component separation approach, or dissection extent, depending on the analytical context.

The present findings directly extend those of our study on reporting completeness [4], which documented that only 51.7% of techniques were fully reproducible on full-text assessment and a mere 3.4% on title/abstract alone. The high rate of polysemy observed here (e.g., TEP mapping to 24 configurations) is largely driven by the very same incomplete specification of mesh type and fixation domains identified in that analysis.

## Limitations

Several limitations should be acknowledged. First, the dataset was derived exclusively from PubMed-indexed literature and does not represent exhaustive bibliographic coverage. The use of descriptive saturation as a stopping criterion, while appropriate for the study objectives, means that rare configurations may have been missed. Second, the analysis focuses on structural diversity rather than clinical effectiveness and cannot determine whether specific configurations are associated with superior outcomes. Third, the five-domain framework is intentionally simplified; additional technical variables not captured here may be relevant to clinical outcomes. Fourth, the label–configuration discordance analysis was restricted to labels explicitly present in the source material and should not be interpreted as a comprehensive linguistic inventory of all labels in use. Finally, although the five-domain framework deliberately omits secondary modifiers (defect size, fascial closure, component separation), these can be added as optional extensions once the core A–H–M–T–F model is widely adopted.

## Implications and future directions

The findings suggest that conceptualizing hernia repair techniques as modular domain combinations rather than eponymous procedures may improve both the clarity and comparability of surgical reporting. A domain-based classification system could serve as a standardized reporting framework for future trials, facilitate the development of machine-readable surgical registries, and support more rigorous pooling of data in systematic reviews and meta-analyses. Integration of this approach with existing reporting guidelines) and registry platforms represents a logical next step.

## Conclusion

Abdominal wall hernia repair techniques are more completely represented when familiar labels are supplemented by structured operative domains than when they are defined by labels alone. The current label-based nomenclature—encompassing acronyms, eponyms, and narrative descriptors—introduces substantial terminological redundancy and classification uncertainty that may undermine the comparability and reproducibility of surgical research. Domain-based representation offers a structured, reproducible, and machine-readable complement to existing labels that may improve reporting quality, facilitate comparative analyses, and support the development of standardized surgical classification systems.

## Supplementary Information

Below is the link to the electronic supplementary material.

Supplementary file1 (DOCX 20 KB)

## Data Availability

The full dataset of extracted technical configurations and label mappings is available from the corresponding author upon reasonable request.
