# Supplementary material for: Beyond acronyms: a domain-based structural analysis of abdominal wall hernia repair techniques in the contemporary literature
Source: Surg Endosc. 2026 Aug 4;40(11):9507–23. doi: 10.1007/s00464-026-13254-x (PMC13755000; doi:10.1007/s00464-026-13254-x)
Supplement: Supplementary file 1 [file 464_2026_13254_MOESM1_ESM.docx]

**SUPPLEMENTARY MATERIAL**

**Supplementary Table S1. Complete configurations for polysemous labels (labels mapping to more than one five-domain configuration).**

*For each polysemous label, all distinct five-domain configurations identified in the extracted dataset are listed. Domain codes: Approach (o = open, l = laparoscopic, e = extraperitoneal, rl = robotic); Hernia (G = groin, V = ventral, I = incisional, S = spigelian, P = parastomal, U = umbilical); Mesh position (R = retromuscular, P = preperitoneal, I = intraperitoneal, O = onlay, X = no mesh); Mesh type (Sp = synthetic permanent, Sa = synthetic absorbable, Ba = biologic); Fixation (S = suture, Tp = permanent tacker, Ta = absorbable tacker, G = glue, A = self-gripping, X = no fixation). "?" indicates domain not reported in the source manuscript.*

| **Label** | **N** | **All Configurations Identified** |
| --- | --- | --- |
| **TEP** | **24** | e-G???; e-GP??; e-GPSp?; e-GPSpTp; e-GPSpTa; e-GPSpX; e-GPSpS; e-GPSpTp/Ta; e-GPSpA; e-G?SpTp; e-G?SpTa; e-GRSp?; e-GRSpS; e-GRSpX; e-G?Sp?; and 9 additional partially specified variants |
| **TAPP** | **18** | l-GP??; l-GPSp?; l-GPSpG; l-GPSpTa; l-GPSpTp; l-GPSpX; l-GPSpS; l-GPSpA; l-GP?G; l-GP?Ta; l-GP?Tp; l-GP?S; l-GPBaS; l-GPSaG; and 4 additional variants |
| **LVHR** | **8** | l-V???; l-VI??; l-VISpTp; l-VISpTp/S; l-VISpS; l-VI?Tp; l-VI?Tp/S; l-V?SpTp/S |
| **IPOM** | **7** | l-VISpTp; l-VI?S; l-VI?Tp; l-VI?Ta; e-SRSpTp/G; l-VISpS; l-VISpTa |
| **eTEP** | **5** | e-GRSpX/S; e-VR??; e-VRSp?; e-VRSpX; e-IRSpS |
| **open repair** | **4** | o-IRSpS; o-IOSpS; o-V???; o-I??? |
| **r-TAPP** | **3** | rl-GP??; rl-GPSpTp; rl-GPSp? |
| **LESS-TEP** | **3** | e-GP?Tp; e-GPSpTa; e-GPSpTp |
| **CTEP** | **3** | e-GPSpTa; e-GPSpTp; e-GP?Tp |
| **Lichtenstein repair** | **3** | o-GRSpS; o-GPSpS; o-GO?S |

*Note: Some apparent multiplicity reflects incomplete domain specification (indicated by "?") in the source reports rather than true semantic polysemy. The table is organized by decreasing number of configurations per label.*
